# Making decisions immediately post-stress: Evidence for dorsolateral prefrontal cortex involvement with an fMRI study

**DOI:** 10.3758/s13415-025-01304-1

**Published:** 2025-05-28

**Authors:** Revati Mulay, Niels Doehring, Negin Javaheri, Peter Erhard, Manfred Herrmann

**Affiliations:** 1 Department of Neuropsychology and Behavioral Neurobiology, University of Bremen, Cognium, Hochschulring 18, 28359 Bremen, Germany; 2 DFG GRK2739/1 Research Training Group: KD2School—Designing Adaptive Systems for Economic Decisions, Bremen, Germany

**Keywords:** Stress, Decision-making, Risk, FMRI, Functional neuroimaging

## Abstract

Identifying neural markers might help to investigate the specific effects of stress on risky decision-making and to understand the behavioral interventions for risk adjustment. It is proposed that stress makes decision-making less deliberate. We employ fMRI to investigate this, with stress and post-stress decision-making occurring during the same fMRI session with a minimal stress-to-decision-making latency and concurrent electrodermal activity (EDA) measurement. In this within-subject study (40 participants, 18 males, *M*_*age*_ = 24.6 ± 3.9 years), participants performed the “decision-making under risk” task twice: immediately following stress induction, and after a control condition. The order of the stress and control conditions was counterbalanced. Stress was induced by asking participants to solve mental arithmetic tasks under time pressure while receiving negative feedback. During the decision-making task, participants chose between a safe and a risky option with monetary incentives. Behavioral and EDA (*n* = 34; due to technical issues) data confirmed that stress induction was successfully implemented. Participants took less risky decisions post-stress than post-control. An fMRI contrast analysis revealed that the right fronto-opercular and the left anterior part of the dorsolateral prefrontal cortex (dlPFC) exhibited significantly lower activation during decisions post-stress than decisions post-control. The results indicate that decisions taken immediately after exposure to the acute stressor are less risky. Furthermore, decisions post-stress are associated with reduced activation in the regions of the dlPFC, possibly leading to lower cognitive control and less deliberate decision-making post-stress. Interventions to increase dlPFC activation might be suitable to improve the decision-making post-stress.

**Supplementary Information:**

The online version contains supplementary material available at 10.3758/s13415-025-01304-1.

## Introduction

Stress is a biological process to adapt and cope with a stressor that is perceived as challenging or threatening (Duque et al., 2022; World Health Organization, 2023). Although stress response evolved to cope with stressors, it may have indirect negative effects on health that are mediated by the stressed individual’s suboptimal decision-making strategies (Starcke & Brand, 2012). An individual can make significantly different decisions and may have different decision-making strategies under stressful compared with nonstressful situations (Yu, 2016). For example, an individual may make a risky decision of ignoring a red traffic light when under stress for being late for an important meeting. Additionally, financial trading, firefighting, or emergency medicine are examples of work situations where risky decisions are regularly made under stress (Anton et al., 2021; Fernández et al., 2010). Thus, many risky decisions in everyday life situations are closely connected with stress (Simonovic et al., 2017; Starcke & Brand, 2012).

 A model of stress and risky decision-making processing was suggested by Yu (2016) with the “stress-induced deliberation to intuition (SIDI)” hypothesis (Yu, 2016). According to this model, stress causes individuals to make more habitual responses than goal-directed choices, i.e., stress-induced deliberation to intuition (Yu, 2016). Furthermore, a recent study discussed various moderating variables (e.g., nature and onset of stressor, type of decision) that influence decision-making under stress in order to propose a comprehensive model of risky decision-making and stress (Sarmiento et al., 2024). Both of these studies suggest cognitive models and provide testable hypotheses that can explain how stress can influence risky decision-making. However, these cognitive models need further investigation to test and refine their propositions (Sarmiento et al., 2024; Yu, 2016). One avenue to advance research in this domain is identifying the specific neural pathways that warrant more in-depth knowledge of stress and risky decision-making processing (Sarmiento et al., 2024). Neuroimaging studies in this field can give additional information on the neural underpinnings of stress as well as risk processing, which cannot be obtained by behavioral studies alone (Phelps et al., 2014; Starcke & Brand, 2012). A comprehensive model, including neural underpinnings of stress and subsequent risky decision-making, may lead to better interventions to improve the quality of decisions post-stress. Thus, in the present study, we aimed to evaluate the neural correlates of both stress and subsequent decision-making under risk with fMRI.

To investigate the neural underpinnings of stress and decision-making, different biological processes involved in stress response need to be considered. Stress induction results in complex endocrine reactions, among them are the activation of the fast-acting sympathetic nervous system (SNS) releasing catecholamines and the activation of the hypothalamus-pituitary-adrenal cortex (HPA) axis resulting in a release of cortisol (Cannon, 1914; Starcke & Brand, 2016).

The SNS response is more immediate and fast-acting compared with the slower-acting cortisol response (Pabst et al., 2013). Activation of the SNS starts immediately after the onset of stress (Kirschbaum et al., 1993; Starcke & Brand, 2016). In contrast, the cortisol response peaks approximately 20 to 40 min after the onset of stress (Dickerson & Kemeny, 2004; Starcke & Brand, 2016), demonstrating that stress responses differ in their biological basis. Previous research has mostly focused on the slower-acting cortisol effects of stress on risky decision-making by measuring decision-making when cortisol levels are at peak. Thus, although the fast-acting SNS effects on decision-making might be relevant to study, they have received less attention in prior studies (Morgado et al., 2015; Pabst et al., 2013; Starcke & Brand, 2016). This fact is further highlighted by Sarmiento et al. (2024) for decision-making under stress and risk. The authors recommend focusing on the precise timing of events in stress-related decision-making studies and suggest that the temporal alignment between the stressor’s onset and duration and the ensuing responses of the HPA axis and the SNS system must be inspected carefully.

Considering this recommendation, in the current study, we aimed to evaluate the effect of the fast-acting SNS stress response on subsequent decision-making under risk using fMRI, with minimal stress-to-decision-making task latency. For this purpose, we induced stress when participants were inside the MRI scanner with the adapted Montreal Imaging Stress Task (MIST; Dedovic et al., 2005) to achieve minimal stress-to-decision-making task latency.

In the present study, we chose a within-subject approach, wherein all participants were subjected to both the stress and control conditions of the MIST in a counterbalanced order. The within-subject design was selected to minimize the impact of inter-individual differences (Chang et al., 2020). Although prior research states certain limitations of counterbalancing in stress research due to the carryover effect of stress and the cortisol response (Porcelli & Delgado, 2009; Wheelock et al., 2016), we implemented counterbalancing to minimize practice/learning and order effects as follows: half of the participants were first subjected to the stress condition, and the other half were first subjected to the control condition, “within the same fMRI session.” Furthermore, the whole experimental duration was kept as close as possible to 20 min to minimize the effects of cortisol peak, particularly for the group that first started with the stress task. To achieve the minimal stress-to-task latency and to focus on the fast-acting SNS effects of stress, the participants had to subsequently perform the decision-making under risk task (i.e., “post-stress” and “post-control”) immediately after each of the stress and control conditions.

With regards to the stress condition, we hypothesized to find activation in brain regions previously linked to stress processing by prior fMRI reviews on this topic (Berretz et al., 2021; van Oort et al., 2017), namely in insula, prefrontal cortex (PFC), inferior frontal gyrus, inferior parietal lobule (IPL), cingulate cortex, and amygdala during the stress condition as compared to the control condition. To confirm stress manipulation and SNS activation, we employed electrodermal activity (EDA) measures as per the recommendations from Gossett et al. (2018), who proposed that skin conductance might be a suitable method to measure stress response in an MRI scanner environment.

The pre-registered research question for the current study was to explore the difference between the neural correlates of decision-making post-stress and decision-making post-control (Mulay et al., 2023). Based on the prior literature and fMRI meta-analysis (Wu et al., 2021), anterior PFC, inferior frontal gyrus, dorsolateral PFC (dlPFC), and dorsal striatum are some of the candidate brain regions involved during decision-making under risk and we expected to also find activations in these brain areas when comparing decision making post-control versus decision making post-stress. However, to the best of our knowledge, there is no study using neuroimaging methods and focusing on this particular contrast.

Prior theories based on behavioral data further suggest that executive and cognitive control mechanisms are reduced post-stress (Starcke & Brand, 2016; Yu, 2016). Therefore, increased effort might be required to counter the reduced control mechanisms and to make decisions post-stress (Gathmann et al., 2014; Speer et al., 2021; Starcke & Brand, 2016). We hypothesized that the need for increased executive control to process decisions after stress will be reflected by higher activation in brain regions that are related to executive functions and decision-making under risk. As mentioned above, some of these areas are the anterior PFC, inferior frontal gyrus, dlPFC, and dorsal striatum (Wu et al., 2021). Hence, we expected higher activation in these brain areas during decision-making post-stress as compared to decision-making post-control.

## Methods

### Participants and data collection

All participants were recruited from the campus population of the University of Bremen, Germany. Participants were recruited through various advertisements made in-person and online between March and August 2023. The sample consisted of 40 right-handed young adults between 18 and 35 years of age with no neurological or psychiatric disorders (18 males, *M* = 24.6 years, *SD* = 3.9 years). Additional inclusion criteria were MRI compatibility and intermediate or conversational knowledge of the German language. All participants were right-handed, as tested by the short version of the Edinburgh Handedness Inventory (Oldfield, 1971), had normal or corrected to normal vision, and signed an informed consent in accordance with the Declaration of Helsinki. Participants were informed in detail about the experimental design and other study instructions without revealing the goal of stress induction. The study was approved by the ethics committee of the University of Bremen.

The sample size in the current study was based on several recommendations from previous studies as well as by considering several further constraints, particularly the limited time and available resources. Because the experiment did not involve a between-group comparison and brain-behavior correlations were not a part of the pre-registered hypotheses, we did not expect inherently lower power for the current study (Poldrack et al., 2017). The recent literature suggests sample sizes of approximately 40 subjects to detect activations in brain regions with a high probability to detect large effect sizes (Geuter et al., 2018). Thus, we collected data from 40 participants, while increasing the “trial sample size” to further enhance the efficiency of the fMRI data as per the recommendations from Chen et al. (2022).

### Experimental design

The experiment consisted of a within-subject design with two paradigms: stress induction adopted from MIST (Dedovic et al., 2005), and subsequent decision-making under risk.

**Fig. 1:**
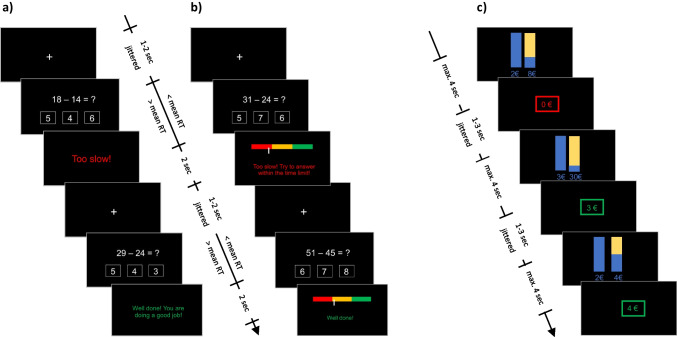
Experimental design of the study for the control (**a**), the stress condition (**b**), and the decision-making under risk task (**c**). In stress and control conditions, the duration of the fixation cross phase was jittered between 1 to 2 s. The maximal allowed response time (RT) was dynamically adapted to the mean RT of the previous trials, with RTs being lower than the mean of previous RTs in the stress condition (**b**) and being higher in the control condition (**a**). In both conditions, the response was followed by a feedback screen displayed for a fixed 2-s interval (English translation of original German text is displayed). In contrast to the control condition, feedback screens in the stress condition consisted of an additional horizontal bar indicating the individuals’ performance. In the decision-making under risk task (**c**), participants had to choose between two vertical bars indicating the probability of wins with a safe option (100%) on the left and risky options (25%, 10%, and 50%, from top to bottom order) on the right. The max. RT for the respective decision was 4 s followed by a feedback screen, risky negative feedback (0€), safe positive feedback (3€), and risky positive feedback (4€)

During both stress and control conditions, participants were asked to solve mental arithmetic tasks involving two numbers ranging from 3 to 50 that had to be subtracted from each other and three possible precalculated responses were displayed on the screen, out of which one was correct. If the participants selected the correct response, positive feedback was displayed on the screen. In case of selecting the incorrect response, negative feedback was displayed on the screen. During the stress condition (60 trials; for details, see Fig. 1b), the maximum time limit to answer each mental arithmetic problem was adapted dynamically in a way that participants always had less time to answer than their mean response time (Berretz et al., 2021; Dedovic et al., 2005). Furthermore, a continuous performance indicator on the screen with a written feedback statement was displayed to the participants during the feedback phase of the stress condition.

This time pressure as well as the continuous performance indicator of negative feedback was expected to result in more severe stress induction as compared to the control condition.

In the control condition, participants solved less difficult mental arithmetic problems, with relatively less time pressure and less harsh negative feedback (60 trials; for details, see Fig. 1a). In contrast to the stress condition, participants had more time to answer each question than their mean response time leading to relatively less time pressure. Furthermore, no continuous performance indicator was displayed during the control condition leading to no continuous evaluative threat.

Both the stress and control conditions were followed by a “decision-making under risk” task which involved a binary choice (Fig. 1c), with both decision-making blocks following the same design. The binary choice consisted of a safe option which always had a 100% chance of winning, and a risky option with varying probabilities of winning. The safe and the risky option had the same expected value (EV). The probabilities of winning or losing were visualized on the screen with blue and yellow bars. The proportion of yellow to blue changed depending on the probability of a win (for details, see Fig. 1c). The risky option had three different probabilities of winning money, either 10% (30 trials), or 25% (28 trials), or 50%(26 trials), adding up to 84 trials in total. The safe option always had a 100% chance of winning. Each block of post-stress and post-control decision-making consisted of 84 trials. All responses were given with a three-button response device.

To account for the carryover effects of stress and to minimize the cortisol response, particularly for the group starting with the stress condition first, we opted for a rather short (~ 20 minutes) experimental duration. Hence, each of the four experimental blocks (stress, post-stress decision-making, control, and post-control decision-making) lasted for approximately 5 minutes. By doing so, we aimed for the cortisol to peak after the whole experiment was finished. Thus, even the group that was first subjected to a stress condition, may not experience the cortisol peak during the experiment, but only after the experiment is over (>20 minutes after the onset of the stressor; Dickerson & Kemeny, 2004; Starcke & Brand, 2016). In order to achieve this, in the current study, all four experimental conditions (stress, post-stress decision-making, control, and post-control decision-making) were incorporated in a single fMRI measurement run with no breaks between the different experimental blocks.

At the end of the experiment, all participants received 5 Euros as a fixed reward. Furthermore, two trials from each decision-making block were chosen randomly and participants received money based on the outcome of those trials. The average total payout was 9.83 Euros (range: 5 to 25 Euros). Participants were informed about the reward scheme prior to starting the experiment.

### Behavioral data and rating scale of stress level

All self-rating data were recorded outside the scanner after the experiment. Participants reported their perceived stress level during the stress and the control condition on an eleven-point scale, ranging from 0: no stress at all to, 10: extreme stress. To measure the math performance for the stress manipulation check, the percentage of correct answers given in time during stress and control conditions was calculated. To measure risky behavior, the percentage of risky decisions was calculated during post-stress and post-control blocks. All of the tests were used as confirmatory as per the pre-registration.

As an exploratory analysis, we employed a mixed ANOVA with counterbalancing order (stress first or control first) as a between-subject factor, condition (post-stress and post-control) as a within-subject factor, and percent of risky decisions as a dependent variable to explore the effects of counterbalancing on risk-taking behavior. To investigate the learning and practice effects during the decision-making tasks, we performed another mixed ANOVA with the counterbalancing order as between-subject factor (stress first or control first), condition (post-stress and post-control) as within-subject factor, and response times during the decision-making task as a dependent variable. Assumptions of mixed ANOVA were checked using Levene’s test for homogeneity of variances. The assumption of normality was checked with the Shapiro-Wilk test. In case of violation of normality, non-parametric equivalents of the parametric tests were used. Behavioral data were analyzed by using R (version 4.1.2; R Core Team, 2021) through RStudio (version 2021.09.2 +382; RStudio Team, 2022).

### EDA measurement in the MRI scanner

During the whole experiment in the MRI scanner, participants' EDA data were recorded. The EDA measurement was conducted using a low-pass filtering EDA device as described by Shastri et al. (2001) with a CED Power1401 data-recording device and a sampling rate of 10 Hz (https://ced.co.uk/). The EDA measurements were temporally synchronized to the scanner's trigger signal and analyzed in Ledalab^®^ V3.4.9. Two pre-gelled, disposable Ag-AgCl electrodes for the EDA recordings were placed on the palmar surface of the left hand of the participants. The data were pre-processed using the adaptive smoothing feature of Ledalab^®^. Afterward, a Butterworth low-pass filter of 0.04 Hz was applied to remove scanner-induced signal distortion. Tonic EDA levels from the entire duration of the stress condition and the control condition were extracted using Continuous Decomposition Analysis (CDA; Benedek & Kaernbach, 2010). We were interested in a slow-varying tonic, rather than an event-related and relatively fast-changing phasic component (Greco et al., 2021). With the approach of CDA, the tonic activity varies over minutes rather than seconds (Benedek & Kaernbach, 2010). As we aimed to measure the overall stress response of SNS during the entire duration of the stress condition (approximately 5 min), we focused on the tonic EDA levels.

This decision was further in line with a study from Greco et al. (2021) who demonstrated the relevance and importance of the tonic component while detecting stress response. Hence, the reported values represent mean tonic activity within the response window of the decomposed tonic component (CDA.tonic, Ledalab^®^ terminology).

### fMRI data acquisition and analysis

Prior to entering the MRI scanner, all participants performed practice trials for solving mental arithmetic tasks with the performance indicator as per the stress condition (15 trials) and for the decision-making under risk task (30 trials).

fMRI data were acquired with a Siemens MAGNETOM Vida Fit^®^ 3-Tesla scanner with a 64-channel head coil and a multiband imaging sequence using a multiband factor of 3 (TR = 1,800 ms, TE = 30 ms, FA = 77°, FOV = 192 x 192 x 138 mm^3^ with a voxel size of 2*2*2 mm, total number of slices = 69). Data processing and analysis were performed with SPM12 (version 7771; Wellcome Department of Cognitive Neurology, 2014, https://www.fil.ion.ucl.ac.uk/spm/software/spm12/). Slice timing correction was achieved according to multiband imaging slice acquisition order. For slice-timing correction, exact slice times were extracted from image DICOM headers, and the first slice was taken as a reference slice. As head motion is the prime source of distortion, data were motion corrected and, in order to address residual motion-induced signal variations, the resulting six motion parameters (translation and rotation) were included as regressors of no interest in the General Linear Model (GLM). For further investigation and quality control for head motion, we analyzed the motion parameters and their first temporal derivative. The net motion between the consecutive volumes was calculated using the formula √ (dmx^**2**^ + dmy^**2**^ + dmz^**2**^ + 58 * (r1^2^ + r2^2^ + r3^2^)) with “dm” corresponding to translational motion parameter and “r” corresponding to rotational motion parameter in x, y, and z planes. Volumes where the net motion exceeded 0.3 were flagged. The maximum number of flagged volumes was under 5% of total volumes for all participants, i.e., at a threshold of 0.3, a maximum of 34 volumes was flagged (for the single participant) out of the total of 715 volumes indicating that head motion did not play a major role during the experiment.

For co-registration, anatomical T1 scans (structural scans) were used with 1 mm^3^ isotropic resolution, and all functional scans were co-registered with the anatomical scans through normalized mutual information. All data were normalized to a standard MNI template and smoothed with an 8 mm Full-Width-Half-Maximum kernel. In order to fully describe the different events of the experiment, 16 regressors of interest and six motion parameters as regressors of no interest were modeled into the GLM. For the mental arithmetic task, response-time dependent regressors for the periods of task solving were added separately for the stress and control condition.

For the decision-making task, the regressors during the period of decision-making were added in the same GLM during post-stress and post-control blocks. Regressors for safe versus risky decisions were modelled separately leading to a 2 by 2 matrix; safe post-stress, risky post-stress, safe post-control, and risky post-control. In this context, safe decisions are the decisions “for” the safe option in that trial, whereas risky decisions are the decisions wherein participants chose the risky option for that trial. All of the decision-making regressors were duration-modulated, response time dependent, and nonparametric. Regressors of the feedback phases during stress, control, and decision-making were added to the model, but they were not used for the subsequent contrast analysis. For further details on the task-based regressors, refer to Table 1 in the supplementary information. The task regressors were convolved with the canonical hemodynamic response function as integrated in SPM12, without derivatives, and a high-pass filter of 600 s was applied. The contrasts were calculated on a whole-brain level with SPM’s *t*-test. Contrasts were first calculated on an individual level and were then entered into the second-level GLM using a one-sample *t*-test. The first contrast of interest was for the stress versus control condition using the regressors corresponding to the time periods of solving mental arithmetic tasks. To evaluate the main effect of stress on the decisions (the main contrast of interest), both regressors “safe post-stress” and “risky post-stress” were contrasted against “safe post-control” and “risky post-control” using a *t*-statistic approach. The cluster correction for these two contrasts was performed according to the nonparametric permutation testing as incorporated via the SPM toolbox SnPM with 5000 permutations without smoothed variance for cluster-level inference (version SnPM13.1.09, https://www.nisox.org/Software/SnPM13/).

**Table 1:** Contrast of stress > control condition of mental arithmetic task solving

Cluster	Extent	x	y	z	Peak *t* score	Cluster size
1	BL lingual gyrus, middle occipital gyrus	6	−84	−10	10.15	39004
		4	−86	−2	8.91	
		−26	−70	−8	8.57	
	BL fusiform gyrus (BA 37)	46	−50	−14	4.77	
		−46	−44	−22	4.65	
	R dorsal insula (BA 13)	40	2	0	4.29	
	BL inferior parietal lobule (BA 39, BA 40)	48	−50	28	3.93	
		50	−36	34	4.57	
		−50	−36	30	4.60	
		−60	−44	26	4.71	
	L cingulate gyrus (BA 31, BA 24)	−8	10	36	4.23	
2	R middle frontal gyrus (BA 10, BA 9/46), R inferior frontal gyrus	40	38	22	5.82	1347
		26	52	16	5.44	
		28	36	48	5.74	
3	R precentral gyrus (BA 4, BA 6), R inferior frontal gyrus (BA 44), R insula (BA 13)	54	4	10	5.75	1277
		54	16	24	5.12	
		50	12	30	4.97	
4	L superior frontal gyrus, L middle frontal gyrus (BA 10, BA 9/46)	−22	42	28	5.27	682
		−38	38	14	4.88	
		−24	56	10	4.66	
5	L caudate, L putamen, L thalamus	−12	−6	12	5.03	146
		−10	10	−8	4.30	
		−18	6	−14	3.98	

*Note.* Cluster-defining threshold = 3.6 as per nonparametric permutation testing, *p*_uncorrected (voxel level)_ <.0005, *p *_*FWE*corrected (cluster level)_ <.05, *df* = [1, 39]. Cluster size > 146 voxels

No regions showed higher activation in control than stress and only unidirectional effects were found. Cluster size is displayed in number of voxels. The table shows three local peaks for each cluster. For cluster 1, the first three peaks are local peaks, and in addition to those, as the cluster size is large (~30,000 voxels), local peaks for each anatomical region are also displayed for informative purposes. Note that we used a multiband imaging sequence with a multiband factor of 3 and a voxel size of 2-mm isometric voxels which might have an impact on large cluster size. Peak MNI coordinates are displayed for each cluster. Brainnetome atlas-based Brodmann areas were used for determining the cluster extent and parcellation. L = left; R = right; BL = bilateral

### Pre-registration and minor changes in data analysis

The study was pre-registered on the PsychArchives in July 2023 before starting the data analysis (10.23668/psycharchives.12967). The above-mentioned information on regressors and contrast construction can also be found in the pre-registration.

Although the majority of the analyses were conducted exactly as per the pre-registered template, a deviation from the pre-registration template must be reported. According to the recommendations from Poldrack et al. (2011), the smoothing was first performed with a 6-mm kernel as an initial starting point and was specified in the pre-registration. However, for the detection of larger clusters with smaller effect sizes, a higher kernel was used (Poldrack et al., 2011). The decision to change the pre-processing kernel was made “before” finalizing the results of the second-level GLM model. Hence, the results reported below are with an 8-mm smoothing kernel.

## Results

### Confirmatory analyses for the stress and the decision-making conditions

No participants had to be excluded from the behavioral data analysis as per the data exclusion criteria specified in the pre-registration.

The whole experiment took approximately 20 min (range 18–23 min, *M* = 21 min, *SD* = 1.03 min, *n* = 40) with the stress condition lasting for approximately 5 min (range 4–6 min, *M* = 5.21 min, *SD* = 0.27 min). The exact duration was dependent on the response times of the participants.

**Fig. 2:**
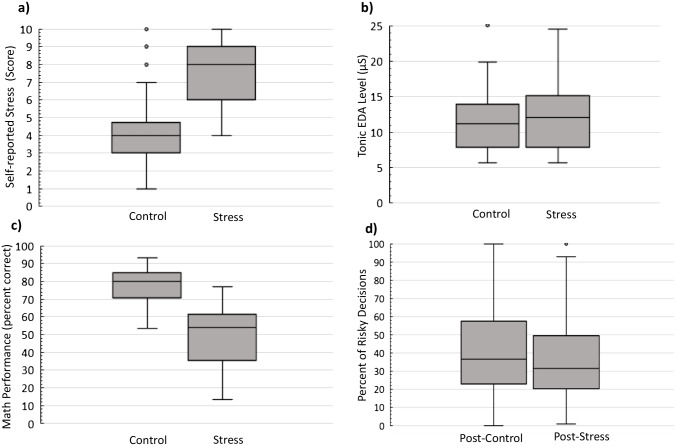
** a**) Participants’ self-reported stress rating on a scale of 0 to 10 (*p* < .0001, *Z* = −5.37). **b**) Tonic electrodermal activity level (EDA, in microSiemens, µS) during control and stress conditions (*p *= *.*02, *Z* = 2.49). **c**) Math performance (percent correct) during control and stress (*p* < .0001, *Z* = −5.51). **d**) Decision-making behavior demonstrated by percent of risky decisions during post-control and post-stress (*p* = .028, *Z* = −2.2)

Data derived from the self-reported stress scale showed that participants reported significantly higher stress levels for the stress condition compared with the control condition (*M*_*stress*_ = 7.6, *SD* = 1.6; *M*_*control*_ = 4.1, *SD* = 2.2). Because these data were not normally distributed, we performed a two-tailed Wilcoxon-signed rank test as a nonparametric equivalent of paired *t*-test (*Mdn*_*stress*_ = 8, *Mdn*_*control*_ = 4, *n* = 39, *z* = −5.37, *p* < .0001, *r* = 0.9; Fig. 2a). Furthermore, participants’ math performance as measured by the percentage of correct answers was significantly lower during the stress condition compared with the control condition (*M*_*stress*_ = 49.7, *SD* = 16.3; *M*_*control*_ = 77.5, *SD* = 10.7; *t(39)* = 16.4, *p* < .0001, *z* = −5.51, *d* = 2.6; Fig. 2c; the median is displayed in box plots). Participants took less risky decisions post-stress (*M* = 37.1, *SD* = 26), than post-control (*M* = 40.9, *SD* = 26.6). This difference was statistically significant as demonstrated by a two-tailed Wilcoxon signed rank test (*Mdn*_*post-stress*_ = 31.5, *Mdn*_*post-control*_ = 36.5, *n* = 39, *z* = −2.2, *p* = .028, *r* = −0.4, Fig. 2d).

### The effect of counterbalancing on decision-making conditions

The mixed ANOVA revealed a significant main effect of counterbalancing order on response times during decision-making. Participants who performed the stress task first were slower during decision-making (*M*_*post-control*_ = 1.02, *SD*_*post-control*_ = 0.32; *M*_*post-stress*_ = 1.16; *SD*_*post-stress*_ = 0.39; in seconds) as compared to participants performing the control task first (*M*_*post-control*_ = 0.99, *SD*_*post-control*_ = 0.17; *M*_*post-stress*_ = 0.80, *SD*_*post-stress*_ = 0.16; in seconds). Thus, counterbalancing order had a significant effect on response times (*F*_*(1,38)*_= 5.42, *p *= .02, *np*^*2*^ = .12). Furthermore, there was a significant effect of interaction revealing that both groups were faster in their responses during the second block of decision-making (*F*_*(1,38)*_ = 34.63, *p* < .001, *np*^*2*^ = .48). “Condition” (post-stress decision-making or post-control decision-making) did not have a significant effect on the response times (*F*_*(1,38)*_ = 0.97, *p* = .33, *np*^*2*^ = .02).

A mixed ANOVA with the percentage of risky decisions as the dependent variable showed a significant main effect of “Condition” (post-stress decision-making or post-control decision-making), indicating a lower percent of risky decisions made post-stress (*F*_*(1,38)*_ = 5.85, *p* = .02, *np*^*2*^ = .1). The other effects did not become significant (counterbalance *F*_*(1,38)*_ = 2.43, *p* = .13, *np*^*2*^ = .06; counterbalance*condition *F*_*(1,38)*_ = 0.11, *p* = .74, *np*^*2*^ < .01). Hence, counterbalancing order did not influence the risky decision-making behavior.

### EDA sensor data

The tonic EDA levels (in microSiemens, µS) from the stress and the control block (CDA.tonic; Ledalab^®^ terminology) were compared. Due to technical reasons during the measurements, the EDA signal from six participants could not be measured, thus the analyses included 34 participants. As the assumption of normality of tonic EDA in each block was violated as per the Shapiro-Wilk tests, we performed the Wilcoxon signed-rank test. The results revealed that the tonic EDA level during the stress condition was significantly higher than during the control condition (Fig. 2b; *Mdn *_*stress*_ = 12 µS, *Mdn *_*control*_ = 11.1 µS, *n* = 34, *z* = 2.49, *p* = .013, *r* = .4, *W* = 152).

### fMRI data

#### Missing data, exclusion of participants & exploratory analyses

In the decision-making task, five participants exhibited a consistent response pattern, either almost always choosing the safe option (*n* = 3) or almost always choosing the risky option (*n* = 2). As outlined in the *Pre-registration* and *Methods* sections, the four regressors for the decision-making task (safe post-stress, risky post-stress, safe post-control, and risky post-control) could not be extracted separately for these five outlier participants.

Thus, a one-sided contrast was manually calculated using only two regressors either only for safe or only for risky regressors, and was later added to the remaining 35 participants for second-level analyses. Hence, all confirmatory fMRI analyses are reported with 40 participants.

Furthermore, we performed two additional exploratory analyses evaluating the negative feedback phases during stress versus during control condition, and decision-making phases during safe versus during risky decisions. The results of these exploratory analyses can be found in the supplementary online information.

#### Contrasting the stress and control conditions

The contrast analysis of the stress and control conditions during the periods of solving the mental arithmetic task was performed to check for the direct effects of stress manipulation. The analysis revealed significantly higher activation during stress than during control condition in several clusters, including in the following regions: dlPFC; bilateral middle frontal gyri (BA 9, BA 46, BA 10), precentral gyrus (BA 4, BA 6), bilateral inferior parietal lobule (IPL; BA 39, BA 40), left caudate nucleus and left putamen, bilateral fusiform gyrus (BA 37) and left cingulate cortex (BA 31, BA 24). Additional information can be found in Table 1.

#### Contrasting the decision-making conditions

**Fig. 3:**
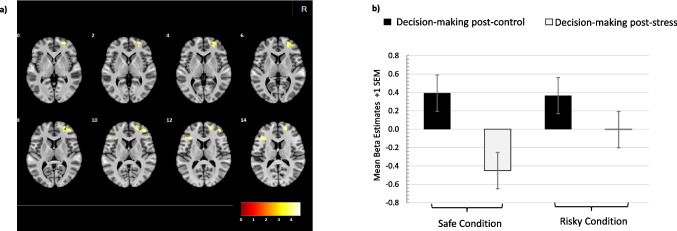
**a**) fMRI contrast of decision-making post-control > decision-making post-stress (cluster size 110 voxels*, p *_*FWE corrected (cluster level)*_* < *.05*, df* = [1, 39] as per nonparametric permutation testing. Right frontal opercular and left middle frontal clusters show higher activation during decision-making post-control compared with decision-making post-stress. Overlayed on MNI152_2009 using AFNI_22.1.06 Antoninus Pius. Color scale indicates *t* values. **b**) Beta estimates from the 8-mm sphere centered at the peak activation of the right frontal clusters with MNI co-ordinates (20 54 14). For both safe (*p* < .001, *t*_[37]_ = 4.8) and risky conditions (*p* = .017, *t*_[36]_ = 2.5), we found significant differences between decision-making post-control and post-stress

Regressors corresponding to decision-making post-stress were contrasted against regressors corresponding to decision-making post-control. The areas of the dlPFC (bilateral middle frontal gyri; BA 10/46 and BA 9/46) exhibited reduced activation post-stress than post-control. Information on statistics and MNI coordinates of the clusters can be found in Fig. 3a and Table 2. Contrary to our hypothesis, we did not find significant differences of activation in other hypothesized regions, such as the inferior frontal cortex or dorsal striatum, for the decision-making contrast. Results from further contrasts for the stress and decision-making task are provided as supplementary online material.

**Table 2:** Contrast of decision-making; decisions post-control > decisions post-stress

Cluster	Extent	X	y	z	Peak *t* score	Cluster size
1	R middle frontal gyrus (BA 10, BA 46)	20 40 26	54 50 56	14 8 2	4.71 4.34 4.28	437
2	L middle frontal gyrus (BA 46, BA 9)	−40 −32 −40	26 26 32	16 12 24	4.54 4.45 4.36	216

*Note.* Cluster-defining threshold = 3.6 as per nonparametric permutation testing, *p*_uncorrected (voxel level)_ < .0005, *p *_*FWE*corrected (cluster level)_ < .05, *df* = [1, 39]. Cluster size > 110 voxels

No regions showed higher activation in post-stress than post-control and only unidirectional effects were found. Cluster size is displayed in number of voxels. The table shows three local peaks for each cluster. Brainnetome atlas-based Brodmann areas were used for determining the cluster extent and parcellation. For reporting the extent, only regions with more than 9% overlap with the total cluster size are shown. Peak MNI coordinates are displayed. L = left; R = right

#### Analysis of decision-making under risk

To explore the specific effects of stress on the post-control and post-stress phases of decision-making, we extracted beta estimates for a region of interest (ROI) based on the above-mentioned decision-making contrast. The beta estimates were extracted using the peak MNI co-ordinates [20, 54, 14] for the right dlPFC and a sphere of 8 mm. Beta estimates were extracted for all decision-making regressors: “safe post-stress”; “risky post-stress”; “safe post-control”; and “risky post-control” and then averaged for the defined ROI for each participant (Fig. 3b). As the whole brain contrast was significant, for both safe (*p* < .001, *t*_*(37)*_ = 4.8) and risky conditions (*p* = .017, *t*_*(36)*_ = 2.5), we also found significant differences between decision-making post-control and post-stress.

## Discussion

The purpose of the study was to assess the neural correlates of decision-making under risk immediately following an acute stressor. Stress induction was successfully implemented as demonstrated by a self-reported stress rating scale and EDA data, with higher EDA in the stress condition presumably reflecting SNS activation. Additionally, as hypothesized, several brain areas (insula, dlPFC, inferior frontal gyrus, inferior parietal lobule, cingulate gyrus) that are involved in stress processing (van Oort et al., 2017) showed higher activation during the stress condition as compared to the control condition additionally proving the successful stress manipulation that will be discussed in this chapter (see Table 1 & supplementary material). With respect to the decision-making under risk task, areas of the dlPFC exhibited reduced activation during decision-making post-stress as compared to post-control. Hence, the reduced activation of the regions of the dlPFC was associated with reduced risk-taking behavior during post-stress decision-making.

### Behavioral data

Previous literature suggests that stress may lead to more effortless and less deliberate decision-making (Yu, 2016). In the present study, the safe option can be characterized as the option requiring less cognitive effort, because it always had a 100% chance of winning as compared to the risky option where participants had to choose between different probabilities of winning (either 10%, 25%, or 50%). As participants chose the risky option less frequently during post-stress decision-making, our results are in line with previous research suggesting more effortless decision-making post-stress. The behavioral data are also in line with Porcelli & Delgado (2009) who employed a cold-pressor task for a 2-min duration and subsequently measured financial risk-taking in the gain domain similar to the binary lottery task used in our study. The results revealed that participants made significantly fewer risky decisions due to stress as compared to a non-stress condition, which ties in well with the present data where participants also made less risky decisions post-stress. Another pharmacological study administering propranolol, a drug known to diminish SNS activity, revealed increased risk-taking behavior (Rogers et al., 2004; Sarmiento et al., 2024). Thus, it can be concluded that in the present experiment increased SNS activation during stress led to reduced risk-taking behavior post-stress.

Our data are in contrast with the meta-analysis by Starcke & Brand (2016), suggesting that stress leads to an increased risk-taking tendency during decision-making. This meta-analysis suggests that this effect is particularly strong when increased risk-taking is disadvantageous.

In our study, we cannot consider risk-taking as particularly disadvantageous or beneficial because both the safe and risky options had the same EV, which might account for the difference between the present results with the meta-analysis by Starcke & Brand (2016).

To further explore the presumed SNS effects of stress on risky decision-making, we calculated the percentage of risky decisions for both of the counterbalanced groups. We observed that risk-taking decreases after stress for both counterbalancing groups. Our results are in alignment with Pabst et al. (2013), who demonstrated that risk-taking tendency decreases between 5 and 18 min after stress onset and “increases” after cortisol peaks approximately 20 to 40 min after stress onset. Since the duration of our whole experiment was around 20 min, even the counterbalanced group that first started with the stress condition might have experienced the cortisol peak only after the experiment was finished (Dickerson & Kemeny, 2004; Starcke & Brand, 2016). Therefore, we expect that the present experimental design minimized the effects of the slower-acting cortisol stress response by keeping the experiment duration rather short, and thereby focusing more on the fast-acting SNS stress response. However, this claim should be interpreted with caution as there might have been an increase in cortisol levels without reaching the peak.

### fMRI data

The fMRI results from the stress versus control contrast during the period of solving mental arithmetic tasks are largely in line with our hypothesis and prior fMRI literature examining the stress response by reviewing the studies employing MIST (Berretz et al., 2021; van Oort et al., 2017). We link these findings specifically to the study by Dedovic et al. (2009), who introduced an event-related MIST paradigm to investigate the effects of solving mental arithmetic during stress. Similar to Dedovic et al. (2009), we found increased and widespread activations in several brain regions that are involved in cognitive and emotion processing, i.e., dlPFC, insula, and cingulate cortex during stress (Dedovic et al., 2009).

We found significantly higher activation in the cingulate cortex, an area that has been discussed in stress processing in relation to the Salience Network (SN; van Oort et al., 2017; Hermans et al., 2014; Vriens, 2020). Furthermore, we found significantly higher activation in regions of the IPL. This finding agrees with the review data from van Oort et al. (2017), showing that several regions of the Default Mode Network (DMN), including IPL, are up-regulated in stress processing. Activation in the basal ganglia has been found in previous neuroimaging studies measuring stress response and has been associated with muscle tension and fight or flight response (Berretz et al., 2021; Cannon, 1939). The current data showing higher activations in the caudate and putamen during stress corroborates this finding.

Similarly, activation in motor areas and precentral gyrus has also been documented in studies employing MIST according to the meta-analysis by Berretz et al. (2021). Contrary to our hypothesis, we did not find significantly higher activity in the amygdala during stress as compared to the control condition during mental arithmetic task solving. The reason for these findings might be linked to our stress induction paradigm that was adapted from MIST. Relative to the other stress induction paradigms used in fMRI studies, such as the Aversive Viewing Paradigm (Henckens et al., 2009) or scanSTRESS (Lederbogen et al., 2011), changes in activity in the amygdala were less prevalent in the MIST, according to the fMRI meta-analysis by Berretz et al. (2021).

In addition to DMN areas, higher dlPFC activation during stress might be linked to the cognitive load of solving mental arithmetic tasks (Berretz et al., 2021). Specifically, the dlPFC (BA 9 and BA 46; Jung et al., 2022; Petrides, 2005) exhibited higher activation during stress than during control condition (see Table 1). Our finding of anterior dlPFC activity is in line with a recent functional near-infrared spectroscopy (fNIRS) study that showed consistently higher dlPFC activity during standard stress induction paradigms, such as the TSST or socially evaluated cold pressor test (SECPT; Schwabe et al., 2008; Meier & Schwabe, 2024). This finding of increased dlPFC activation during stress may appear in contrast with studies suggesting that catecholamine release interferes with PFC function (Arnsten, 2009) and affects large-scale networks, such as the Executive Control Network (ECN), including the dlPFC (Hermans et al., 2011; Hermans et al., 2014; Meier & Schwabe, 2024). The dlPFC is a major brain area associated with executive functioning and a key node in the ECN (Nee et al., 2013; Starcke & Brand, 2016). It has been proposed that the ECN will be suppressed when the catecholaminergic effects of stress are particularly strong (Hermans et al., 2014). Despite this finding, we found increased activation in regions of ECN, such as the dlPFC, while solving mental arithmetic tasks under stress. This conflicting data might be because some studies that show suppression of ECN employed pharmacological interventions, that are different from the current study’s stress condition, involving mental arithmetic tasks with cognitive effort (Meier & Schwabe, 2024). The mental arithmetic problems in the stress condition were comparatively more difficult than those in the control condition, and they had to be solved under strict time pressure, with the threat of receiving harsh negative feedback combined with social evaluation. Hence, the higher activity in dlPFC can be linked to higher cognitive load and stress processing (van Oort et al., 2017). Similar to Meier & Schwabe (2024), we support the idea that the dlPFC might be involved in adapting to the stressor, irrespective of the stress induction protocol.

The higher activity in the dlPFC “during” stress was not sustained during “post-stress” decision-making in the current experiment. We hypothesized that the need for increased executive control to process decisions after stress will be reflected by higher activation in the dlPFC.

Contrary to our hypothesis, we found decreased dlPFC activation during decision-making post-stress. This finding might indicate that the participants were not able to sustain the required cognitive control or executive effort to process the decisions after the stress task was completed. We further link these data to the findings of the fNIRS study by Meier & Schwabe (2024), showing that the increased activation of the dlPFC during stress was transient. The authors found increased dlPFC activation only during the stress task, but not afterward in the post-stress resting phase (Meier & Schwabe, 2024). Thus, in their study, the dlPFC activity was not significantly different in the aftermath of stress between the stress versus the control groups (Meier & Schwabe, 2024). The authors suggested that post-stress differences in the dlPFC activation may be found if this phase has cognitive demands, and not during rest. Our data confirm this hypothesis as participants were required to engage in the cognitive task of “decision-making under risk” in the post-stress phase and increased dlPFC activation during stress was not sustained in the post-stress decision-making phase.

Reduced activation of the dlPFC during decision-making post-stress as compared to post-control can be linked to higher release of catecholamines due to SNS activation immediately after stress (Hermans et al., 2014; Ossewaarde et al., 2011; Qin et al., 2009). Our data showing reduced dlPFC activation during decision-making post-stress are in alignment with prior studies demonstrating that dlPFC activation is suppressed shortly following the stress induction in relation to executive functions. The dlPFC has been suggested to be involved in executive functioning, specifically cognitive control (Bari & Robbins, 2013; Mohr et al., 2010; Wu et al., 2021), which regulates goal-oriented and flexible behaviors (Schonberg et al., 2012; Wu et al., 2021). Inefficient cognitive control through dlPFC inhibition might lead to less fine-tuned adjustments and non-goal-directed choices during decision-making (Yu, 2016). Furthermore, it has been proposed that the dlPFC plays a major role in the development of coping strategies and planning adaptive behavioral responses during decision-making (Sarmiento et al., 2024). In our experiment, participants exhibited risk-averse behavior during decision-making post-stress. This risk-averse behavior associated with reduced dlPFC activation may indicate that participants were unable to process the trade-off between risk and rewards during post-stress decision-making leading to risk aversion (Gowin et al., 2013; Wu et al., 2021). Thus, in accordance with the SIDI model, participants made more safe choices in post-stress decision-making, which can be characterized as their habitual response.

Contrary to our hypothesis, we did not find differences in the post-stress versus post-control decision-making in other brain regions such as the inferior frontal gyrus or dorsal striatum. These findings might be linked to a causal role of dlPFC in the avoidance of risk during decision-making (Fecteau et al., 2007; Obeso et al., 2021).

For example, Obeso et al. (2021) applied repetitive transcranial magnetic stimulation with theta burst stimulation (cTBS) to temporarily interfere with the neural activity of the dlPFC. The results revealed that temporary inactivation of the dlPFC is associated with avoidance of risky choices in the Iowa Gambling Task (IGT). This finding might explain why our data showed differences in activation patterns for post-stress versus post-control decision-making only in the dlPFC but not in other brain regions. In accordance with Obeso et al. (2021), we observed reduced risk-taking post-stress coupled with reduced activation in the dlPFC during decision-making post-stress.

The exploratory analysis of beta estimates from an ROI based on the decision-making contrast revealed the underlying neural signal patterns between post-stress versus post-control decision-making in further detail. For both safe and risky decision-making in the post-control phases, the difference between the beta estimates was rather small. However, this difference became much more pronounced in post-stress decision-making (Fig. 3b). This further indicates that the risk processing post-control was significantly different compared with risk processing post-stress.

## Limitations and directions of future research

### Effects of counterbalancing on the experimental design and results

In our experimental set-up, counterbalancing offered certain advantages, such as compensating for scanner drift and practice, or learning effect. We kept the decision-making under risk task rather simple with known probabilities of winning to minimize the learning effects. Despite this, both counterbalanced groups showed faster response times in the second block of decision-making demonstrating learning or practice effects, which can be accounted for due to counterbalancing. On the other hand, there are some limitations to counterbalancing with the stress condition. This is particularly demonstrated by the analysis of response times during the post-control and post-stress decision-making tasks. The group that started with the stress condition showed higher response times during the decision-making task than the group that started with the control condition first, suggesting a carryover effect of stress. We attempted to design the study in a way that the whole experiment duration was kept rather short before cortisol peak levels were reached (Dickerson & Kemeny, 2004; Starcke & Brand, 2016). However, because we did not use direct cortisol measurements during the experiment, we cannot prove this hypothesis within the present experimental setup.

### Decision-making under risk task

We employed a relatively simple and well-known binary lottery task to assess decision-making under risk. As the EV of both safe and risky options was kept the same and the probabilities of winning were always known to the participants, we cannot conclude that risk-taking in our study was particularly advantageous or disadvantageous. Furthermore, the relevance of risk-taking in the current experimental design might not fully reflect real-life situations, e.g., emergency room or stock trading. In the current experimental design, we aimed to keep the stress condition distinct from the decision-making task as we were interested in studying the immediate aftermath of stress on decision-making. Despite this fact, it should be noted that many real-life situations involve the intermingling of stress and decision-making (e.g., combat situations) where the temporal distinction between stress and decision-making is not clear.

According to the meta-analysis of Starcke & Brand (2016), one of the motivating factors of research on stress-related changes in decision-making is to understand why and under which circumstances stress leads to less advantageous decisions. Thus, future studies might incorporate a similar paradigm by varying the EV or by incorporating probabilities of win that are unknown to the participants (decision-making under ambiguity). Thereby, a distinction between advantageous and disadvantageous decisions can be made more explicit. In contrast to our binary lottery task where participants knew the explicit probabilities of winning, the Balloon Analogue Risk Task (BART), or the IGT, introduces an implicit risk probability, where participants are unaware of the exact chances of winning or losing (Sarmiento et al., 2024). Whether the same behavioral and neural effects emerge when testing our experimental design with BART or IGT remains a question for future research. Another interesting topic in this domain is to introduce our experimental paradigm in other nonfinancial domains, e.g., social incentives.

A further potential topic for future research is to introduce a “recovery period” in the current experimental setup. Thus, participants can be instructed to employ strategies that are proven to elicit dlPFC activation (e.g., working memory tasks) during the recovery period for 1 or 2 min, and it can be evaluated if this intervention increases the dlPFC activation during decision-making post-stress.

## Conclusions

In the present study, we assessed the neural correlates of stress and subsequent decision-making under risk using fMRI. In our experimental design, the stress-to-decision task latency was minimized to focus on the fast-acting SNS/catecholaminergic effects of stress. Our data show that several brain regions, including areas of the dlPFC, exhibited higher activation during the stress-associated solving of mental arithmetic tasks. However, this increased activation was not sustained during the post-stress decision-making. Thus, increased dlPFC activation was present for the transient time period of the stressor, but not during the post-stress decision-making. This finding can be linked to fast-acting catecholaminergic effects in the aftermath of stress on dlPFC. Therefore, our data support the SIDI model (Yu, 2016), indicating that acute stress leads to less deliberate decision-making through dlPFC inhibition (Arnsten, 2009; Hermans et al., 2014; Sarmiento et al., 2024). In our experiment, this behavior can be characterized by a reduced risk-taking tendency and choosing the safe option more frequently during post-stress decision-making.

Most individuals are occasionally or recurrently confronted with acute stressors across life domains (Weber et al., 2022), which can be compared with the short-lasting stressor (around five minutes) in our experiment. Based on our fMRI data, we argue that interventions or strategies targeting dlPFC functions might be the best to alleviate the immediate effects of stress on subsequent decision-making.

An integrative framework for understanding complex interactions between moderating variables of stress and decision-making (e.g., onset and duration of stress, neural reactions to stress, etc.) was recently proposed (Sarmiento et al., 2024). Our study provides experimental evidence in this field and may offer newer insights for improving the quality of decision-making post-stress.

## Supplementary Information

Below is the link to the electronic supplementary material.

Supplementary file1 (DOCX 3605 KB)

## Data Availability

The data sets used and/or analyzed during the current study are available from the corresponding author upon reasonable request.

## Code availability

The analysis and experiment scripts are available from the corresponding author upon reasonable request.
