# Supplementary material for: Making decisions immediately post-stress: Evidence for dorsolateral prefrontal cortex involvement with an fMRI study
Source: Cogn Affect Behav Neurosci. 2025 May 28;25(5):1291–305. doi: 10.3758/s13415-025-01304-1 (PMC12464067; doi:10.3758/s13415-025-01304-1)
Supplement: Supplementary file 1 — Supplementary file1 (DOCX 3605 KB) [file 13415_2025_1304_MOESM1_ESM.docx]

**Table S1**

*Regressors used in the General Linear Model of fMRI Analysis*

| **Regressors for** | **Math Task** | |
| --- | --- | --- |
| **Contrast** | **Stress** | **Control** |
| Mental Arithmetic Task Solving | 1 | 2 |
| Positive Feedback | 3 | 4 |
| Negative Feedback | 5 | 6 |
| **Regressors for** | **Decision-making under Risk Task** | |
| **Contrast** | **Post-Stress** | **Post-Control** |
| Decision for Safe | 7 | 8 |
| Decision for Risky | 9 | 10 |
| Safe Win Feedback | 11 | 12 |
| Risky Win Feedback | 13 | 14 |
| Risky Loss Feedback | 15 | 16 |

**Exploratory results with two additional contrasts**

***Contrasting negative feedback during stress vs. during control condition***

The first contrast analysis was conducted using regressors of negative feedback phase during stress vs. control. The analysis revealed that regions of occipital lobe (lingual gyrus, middle occipital gyrus) were significantly more active during stress compared to control during negative feedback (Figure S1a, supplementary information). This finding further confirms the stress manipulation particularly because participants saw the performance indicator on the screen during stress, but not during the control condition (see experimental design in the main text). Thus, seeing additional information on the screen might have led to increased occipital lobe activation during “stress negative feedback” as compared to “control negative feedback”. Moreover, areas in the pre-frontal cortex (medial and middle frontal gyrus) exhibited reduced activation during stress negative feedback as compared to control negative feedback (Figure S1a, supplementary information). This finding may indicate altered feedback processing during stress as compared to control condition. These results are in alignment with Dedovic et al. (2009b) who incorporated an event MIST design and separated the negative feedback events from the events of solving mental arithmetic tasks. The results of their contrast analysis comparing negative feedback during the stress condition against positive feedback from the stress condition, revealed significantly lower activation in the regions of the dmPFC and dlPFC (medial and lateral orbitofrontal areas), particularly in cortisol responders indicating the roles of these regions for feedback processing during stress (Dedovic et al., 2009).

***Contrasting decision type safe vs. risky***

Secondly, to evaluate the main effect of decision-type, we conducted another contrast analysis contrasting “safe post-stress” and “safe post-control” against “risky post-stress” and “risky post-control” (Figure S1b; supplementary information). The results were largely unidirectional showing that making risky decisions led to overall higher activation as compared to making safe decisions. The identified regions (insula, caudate, putamen, cingulate gyrus, inferior parietal lobule, inferior frontal gyrus, medial frontal gyrus, and middle frontal gyrus) have also been implicated in risk processing according to prior fMRI meta-analyses (Poudel et al., 2020; Wu et al., 2021), further validating our experimental design and results.

**Figure S1a)**

*Brain regions demonstrating significant difference of activation for the fMRI contrast analysis of negative Feedback during stress vs. during control condition*

Figure S1a shows the contrast analysis of negative feedback processing during stress vs. during control condition. In the red clusters, higher brain activation is observed during negative feedback stress condition, blue clusters show higher brain activation during negative feedback control condition. Cluster size > 104 voxels, cluster defining threshold = 3.6 as per non-parametric permutation testing*, p _FWE corrected (cluster level)_ <* .05*, df* = [1,39]. Overlayed on MNI152_2009 template as incorporated in AFNI version AFNI_22.1.06 'Antoninus Pius'. The color scale indicates *T* values. Figure indicates axial slice view with 3 mm spacing.


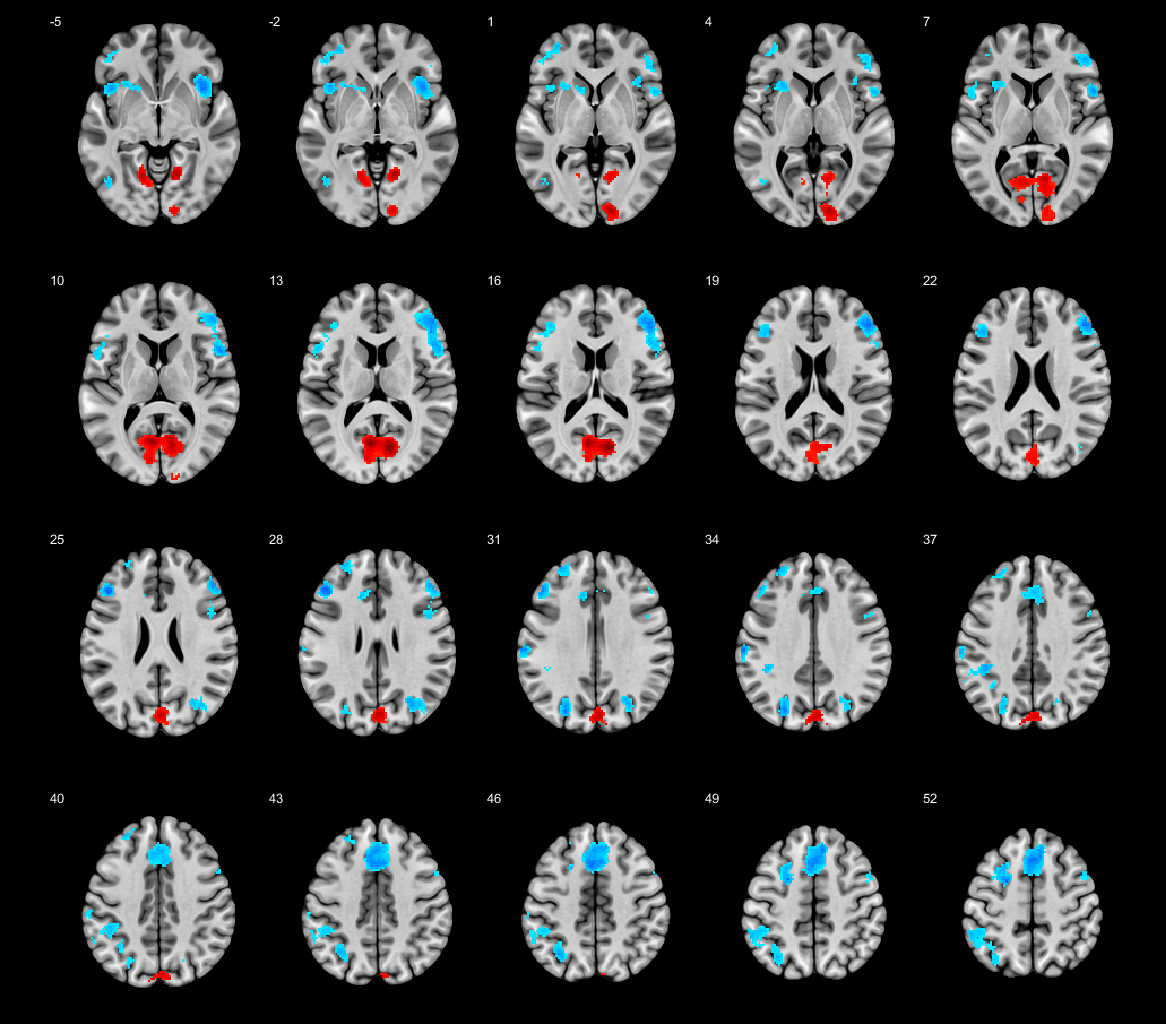

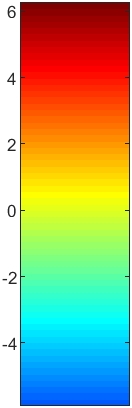


R

Figure S1b shows all regions where higher activation was observed while making a risky decision as compared to safe decision. Cluster size > 93 voxels, cluster defining threshold = 3.6 as per non-parametric permutation testing*, p _FWE corrected (cluster level)_ <* .05*, df* = [1,34].Overlayed on MNI152_2009 template as incorporated in AFNI version AFNI_22.1.06 'Antoninus Pius'. The color scale indicates T values. Figure indicates axial slice view with 3 mm spacing.

**Figure S1b)**

*Brain regions demonstrating significant difference of activation for the fMRI contrast analysis of risky decisions vs. safe decisions*


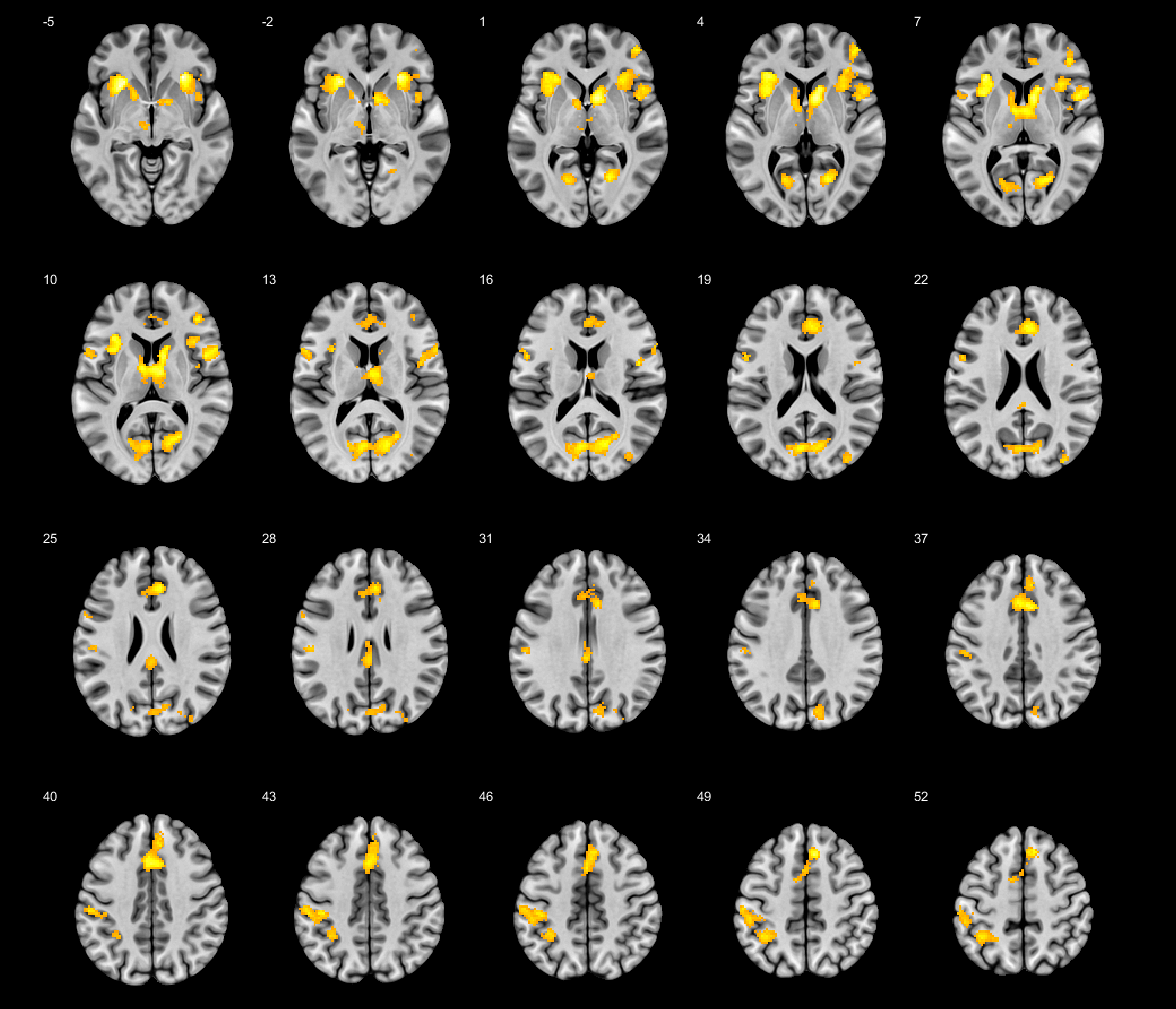

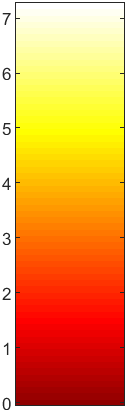


R

R
